# AN INTEGRATIVE LITERATURE REVIEW ON SPIRITUAL NEEDS FROM THE PERSPECTIVE OF OLDER ADULTS LIVING WITH DEMENTIA

**DOI:** 10.1093/geroni/igad104.1267

**Published:** 2023-12-21

**Authors:** Hui Zhao, Augustine Boateng, Francesca Ezeokonkwo, Chad Federwitz, Fayron Epps, Katherine Britt

**Affiliations:** James Madison University, Harrisonburg, Virginia, United States; University of Pennsylvania, Philadelphia, Pennsylvania, United States; James Madison University, Harrisonburg, Virginia, United States; Western Colorado Community College/Colorado Mesa University and Pitkin County Senior Services, Grand Junction, Colorado, United States; Emory University, Atlanta, Georgia, United States; The University of Pennsylvania, Philadelphia, Pennsylvania, United States

## Abstract

Spirituality is a part of successful aging, promoting better sleep, well-being, and cognitive function. Persons living with dementia (PLWD) are reported to have greater needs related to their spirituality (spiritual needs) compared to persons without dementia; however, support for spiritual needs is minimally present or absent in dementia care. Unmet spiritual needs may lead to depression, anxiety, and greater physical pain; thus, addressing the spiritual needs of PLWD is essential to support well-being and inform personalized care. Our integrative literature review explored characteristics of spiritual needs of PLWD and how to support them. ATLA Religion, CINAHL, PsychInfo, PubMed, and SocIndex were searched between 2000 and 2022, resulting in five original research articles meeting inclusion criteria focusing on spiritual needs of PLWD, including PLWDs’ perspectives. Most studies (n=4) were qualitative design, conducted worldwide in various settings, and included mild to moderate stage dementia. Components of spiritual needs covered the importance of connection and meaning for a sense of peace to support their identity and values. Connection to elements of spirituality included religion, meaningful relationships, nature, art, and self. Meaning was connected to past sources and experiences, supported by spiritual and religious activities. A sense of peace is supported through meaningful activities and religious rituals to help them rely on their faith through fear and memory loss. Support for spiritual needs may facilitate meaningful engagement for PLWD, leading to a sense of peace. Healthcare providers may utilize complementary, low-cost holistic care to promote personhood, values, and well-being among PLWD.

